# Supplementary material for: Inhibition of protein tyrosine phosphatase PTP1B function ameliorates pathophysiological deficits in Rett Syndrome
Source: bioRxiv. 2026 Jun 8:2026.06.04.730096. Preprint. [Version 1] doi: 10.64898/2026.06.04.730096 (PMC13278135; doi:10.64898/2026.06.04.730096)

Supplementary Figure 1

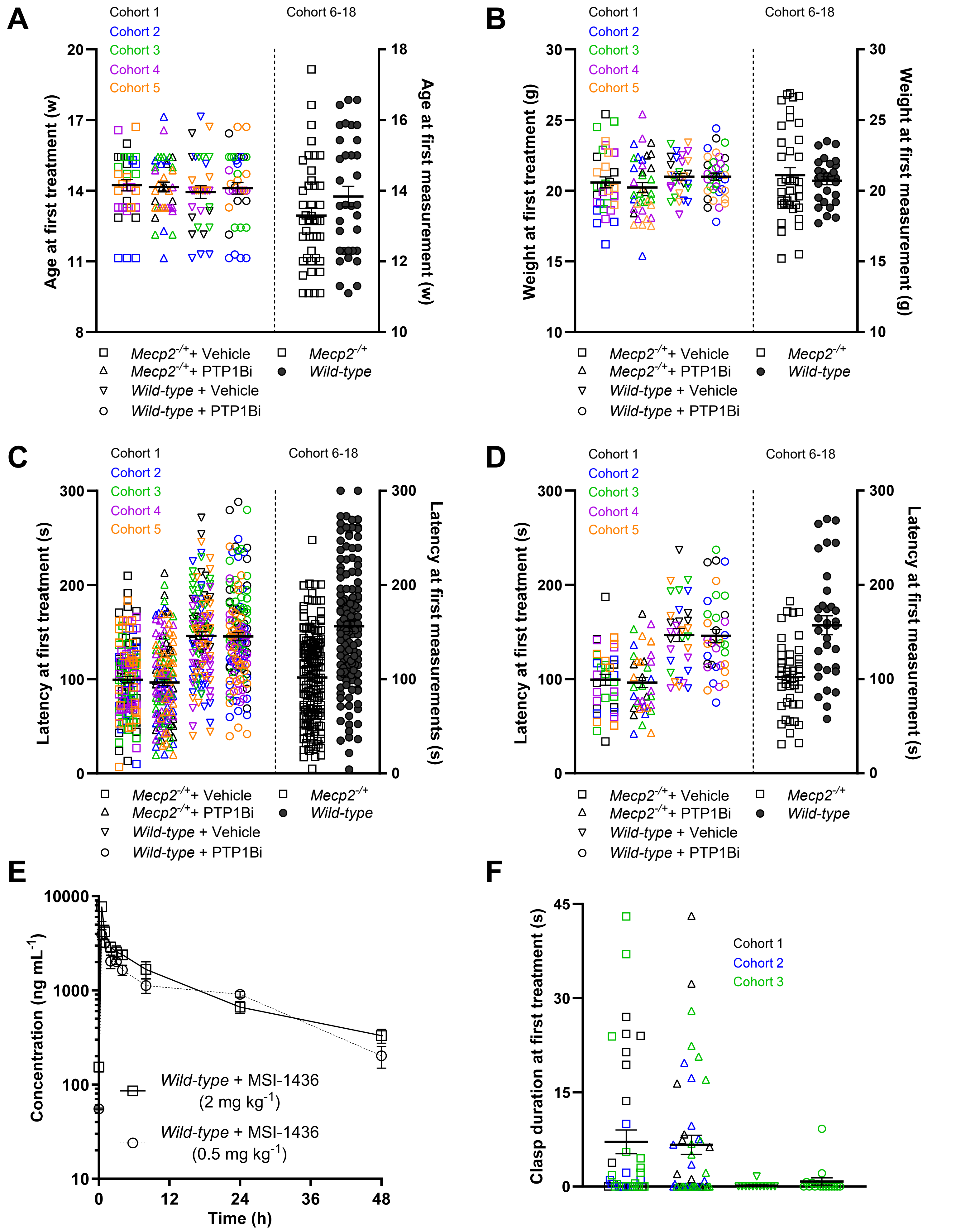

Supplementary Figure 2

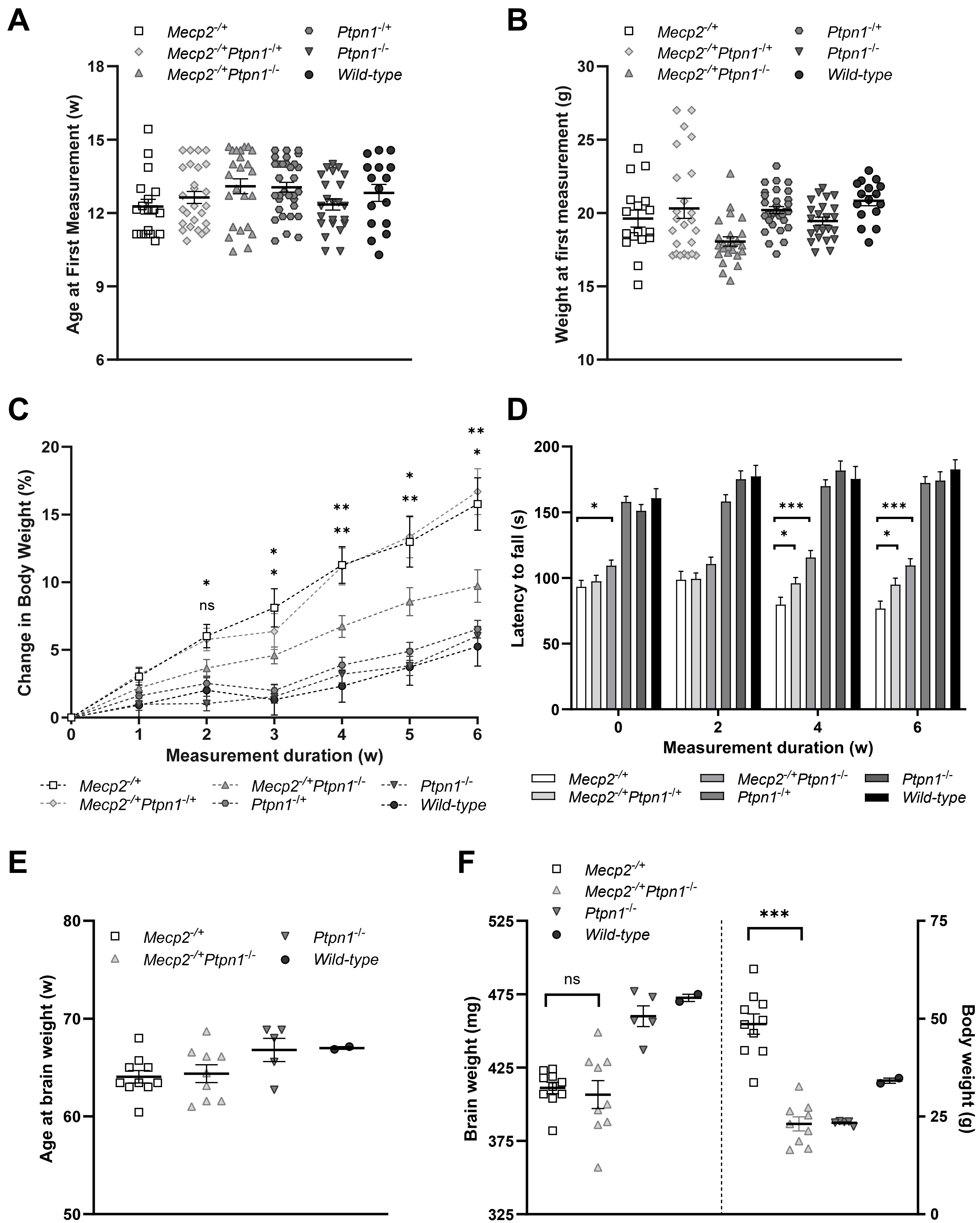

Supplementary Figure 3

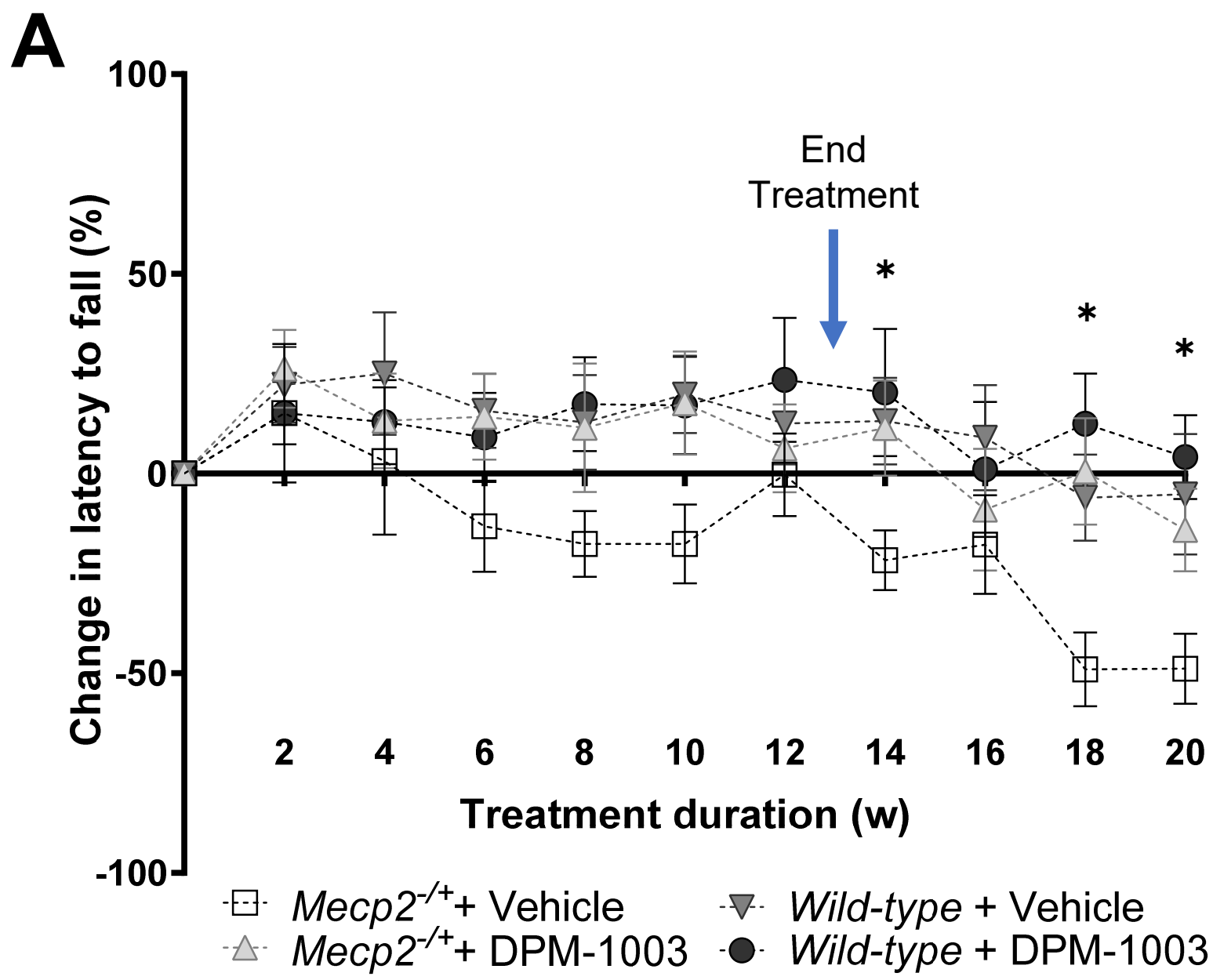

Supplement: Supplementary file 1 [file media-1.pdf]
